# Supplementary material for: Cutting the costs of coastal protection by integrating vegetation in flood defences
Source: Nat Commun. 2021 Nov 11;12:6533. doi: 10.1038/s41467-021-26887-4 (PMC8586143; doi:10.1038/s41467-021-26887-4)
Supplement: Supplementary file 1 — Supplementary Information [file 41467_2021_26887_MOESM1_ESM.pdf]

Supplementary Information on “Cutting the costs of coastal protection by integrating vegetation in flood defences”

Vincent T. M. van Zelst[a*], Jasper T. Dijkstra [a], Bregje K. van Wesenbeeck [a, b], Dirk Eilander [a,c], Edward P. Morris [d,e], Hessel C. Winsemius [a, b], Philip J. Ward [c] and Mindert B. de Vries [a]

[a] *Deltares, P.O. Box 177, 2600 MH Delft, The Netherlands*
[b] *Delft University of Technology, Faculty of Civil Engineering and Geosciences, P.O. Box 5048, 2600 GA Delft,The Netherlands*
[c] *Institute for Environmental Studies(IVM), Vrije Universiteit Amsterdam, 1081 HV Amsterdam, The Netherlands*
[d] *Instituto Universitario de Investigación Marina (INMAR), University of Cádiz, 11510 Puerto Real, Cádiz, Spain*
[e] *Cervest, EC1V 9HX London, The United Kingdom*

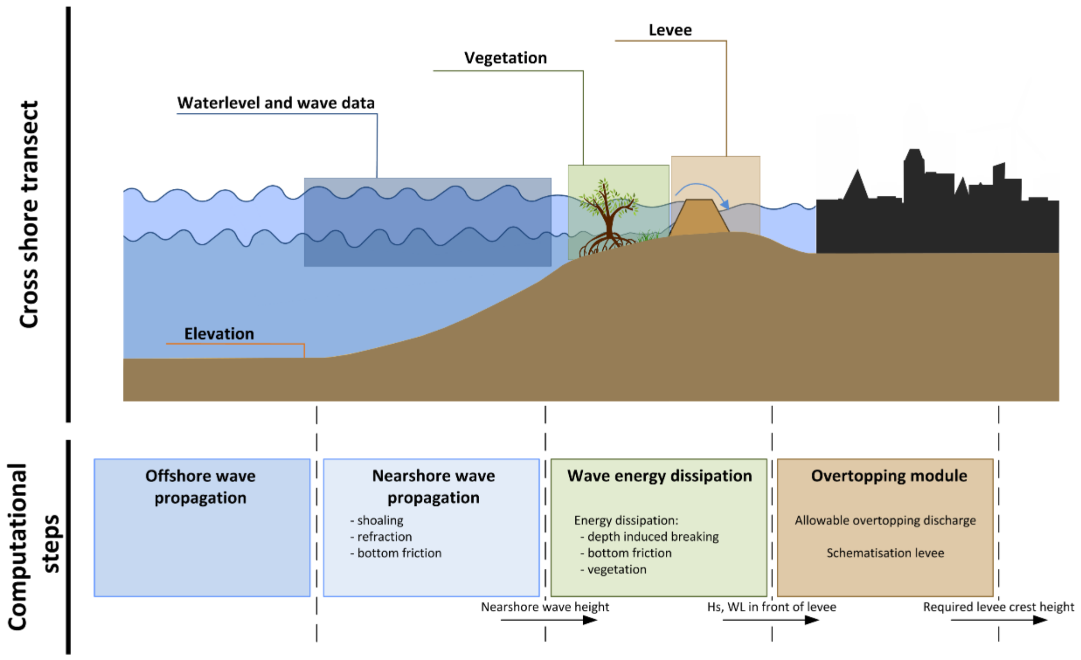

Supplementary Fig. 1 | Coastal properties and computational steps used to assess the role of coastal vegetation in flood hazard reduction. Schematization of the steps taken for each transects to assess flood hazard reduction by coastal vegetation. A deep water wave entering shallow water is affected by the decreasing depth. Closer to shore wave energy is dissipated by bottom friction, wave breaking and possibly due to interaction with vegetation. Eventually the wave can overtop the levee resulting in water entering the hinterland. A maximum allowed wave overtopping discharge results in a required levee height. Calculating the required levee height for a bare and vegetated foreshore results in the reduction in required levee height by coastal vegetation. Figure is created using Inkscape v1.1 (https://inkscape.org/).

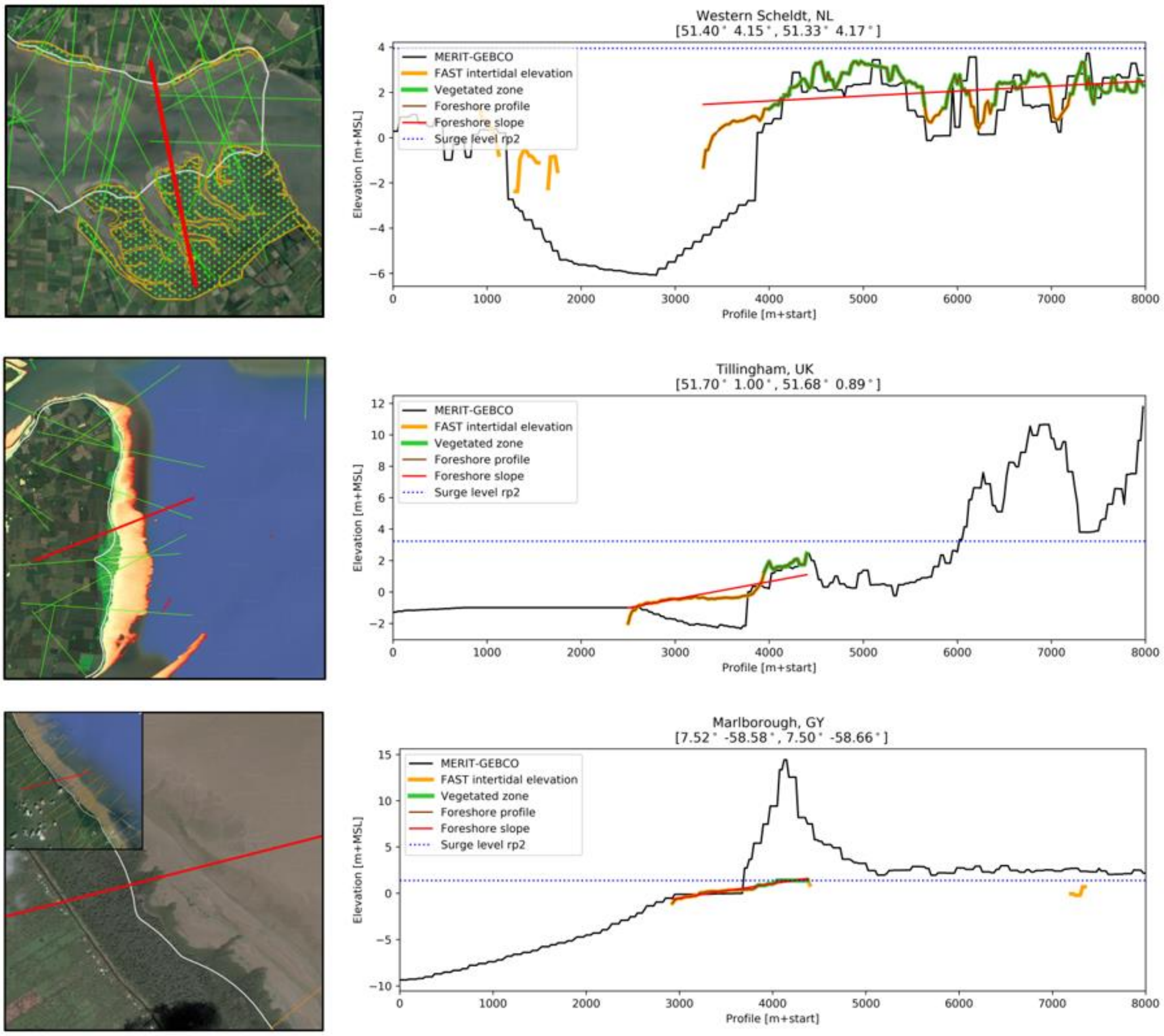

Supplementary Fig. 2 | Example transects. (Top) Transect in the Western Scheldt, The Netherlands, the green dotted polygon shows the extent of the salt marsh map for this location. (Middle) Transect near Tillingham, United Kingdom, the colours in the satellite image represent data from the FAST intertidal elevation map. (Bottom) Transect vegetated with mangroves in Guyana, where the canopies are visible in the MERIT elevation data. Figure topviews (left column) are created with QGIS (http://www.qgis.org/) using satellite images obtained through QuickMapServices QGIS plugin, Map data ©2015 Google. Figure profiles (right column) are created with Python 3.8.10 (https://python.org) using Matplotlib v3.3.4.

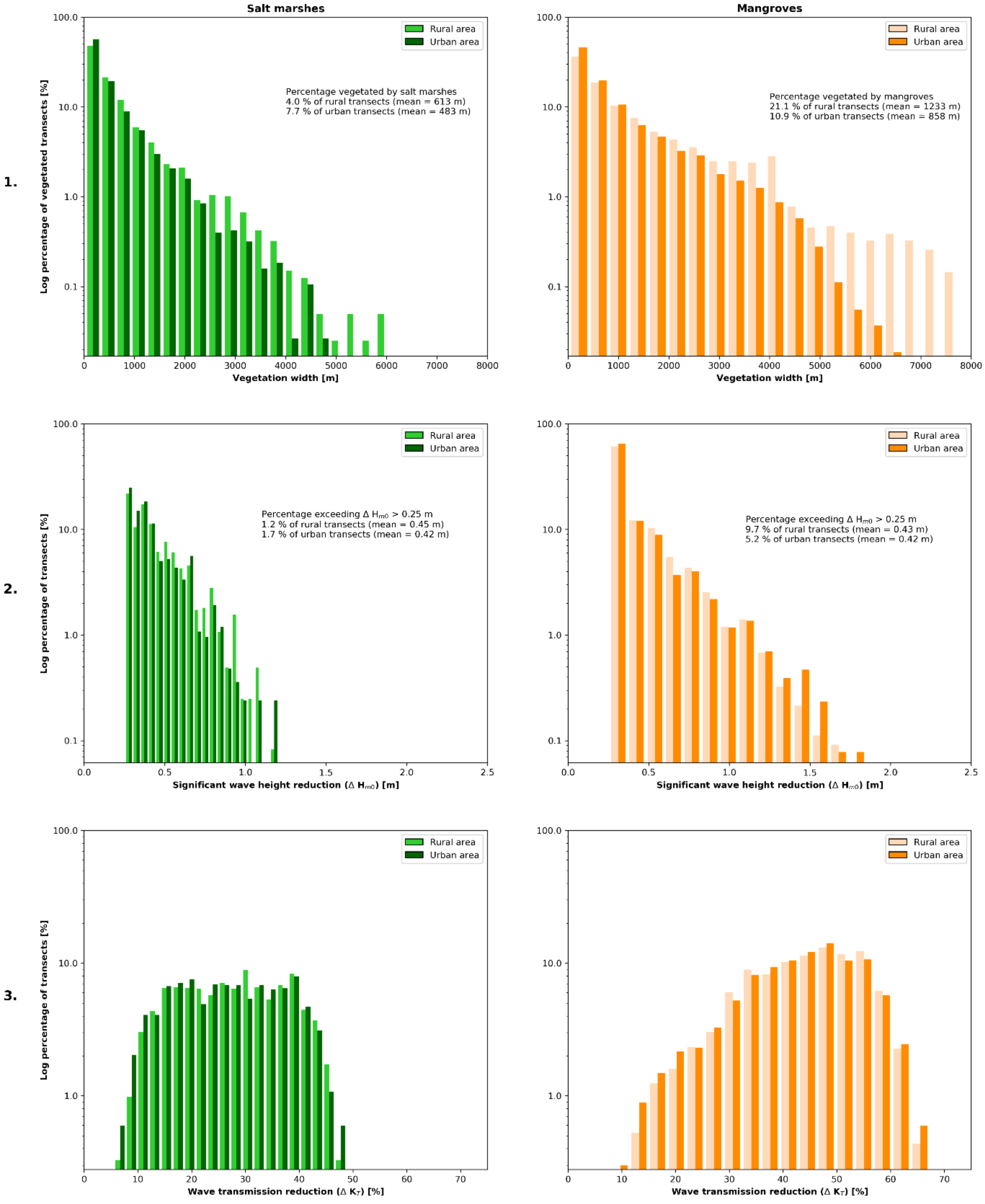

Supplementary Fig. 3 | Coastal vegetation in urban and rural areas that are susceptible to flooding. (1) Distribution of coastal vegetation belt widths over urban and rural areas for salt marshes and mangroves. Vegetation cover in rural areas is far more extensive than in urban areas. **(2)** Histogram showing the distribution of the significant wave height reduction by coastal vegetation in rural and urban areas. **(3)** Histogram showing the distribution of reduced wave transmission by coastal vegetation in rural and urban areas. Reduced wave transmission is defined by the significant wave height reduction divided by the incoming wave height at the front of the vegetated zone.

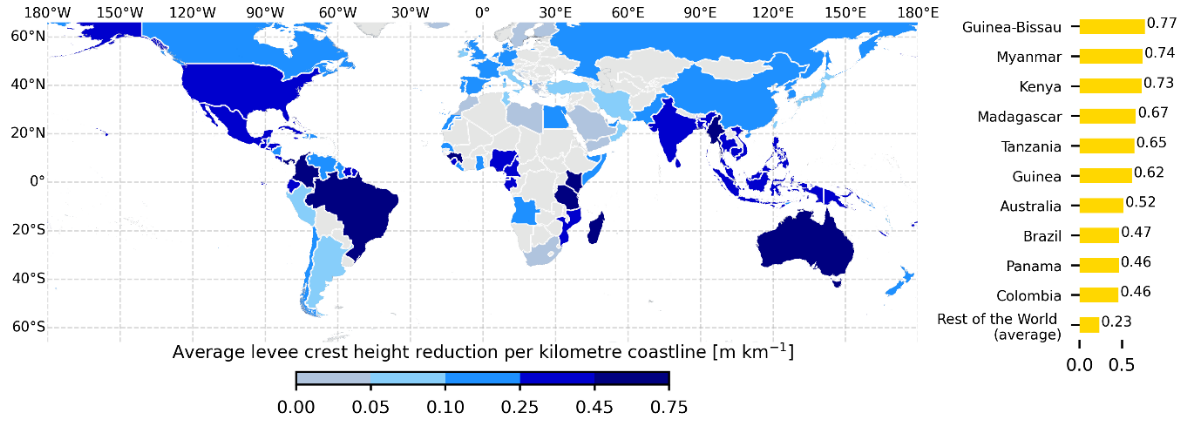

Supplementary Fig. 4 | Country level averages of required levee crest height reduction per kilometre coastline susceptible to flooding. World map showing the average levee height reduction per kilometre coastline per country, due to current vegetation presence. Only countries having a coastline susceptible to flooding of at least 25 kilometres are included. The map is based on storm conditions with a return period of 100 years. Non-benefiting countries are indicated with a light grey colour. Map is created with Python 3.8.10 (https://python.org) using Cartopy (v0.18.0. Met Office UK. https://pypi.python.org/pypi/Cartopy/0.18.0), GeoPandas v0.8.1 (https://geopandas.org), Matplotlib v3.3.4[39] and GADM v2 (https://gadm.org) administrative boundaries.

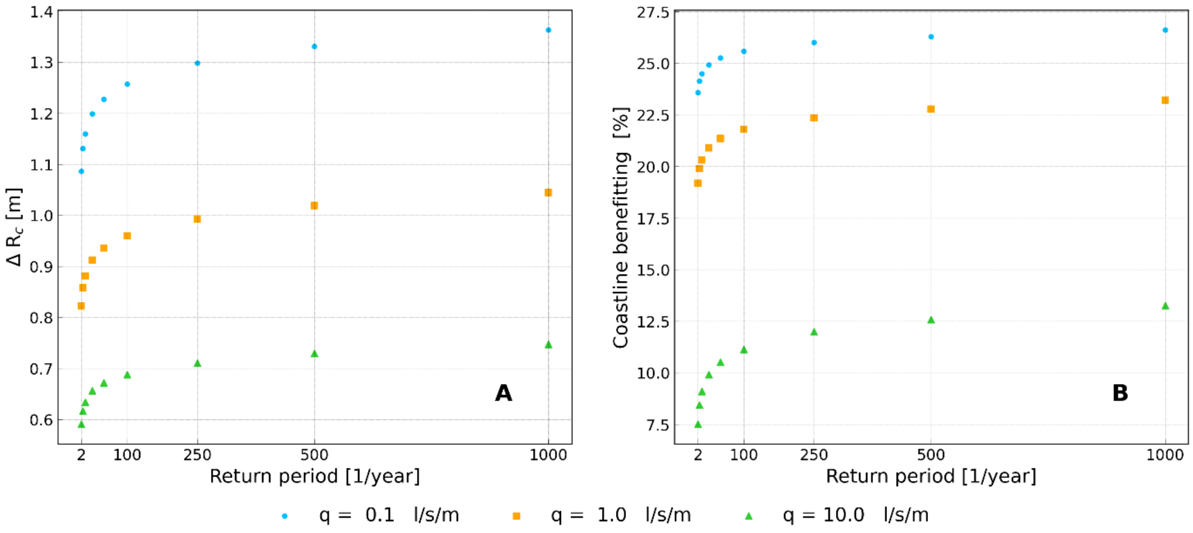

Supplementary Fig. 5 | Required levee crest height reduction and percentage of coastline benefitting due to foreshore vegetation for multiple return periods and critical overtopping rates. (A) The mean reduction of required crest height along the susceptible coastline for nine return periods, i.e. for storm surges and storm conditions with different frequencies of occurrence. The dots, squares and triangles represent the outcomes for the three different critical overtopping rates. **(B)** The percentage of the susceptible coastline benefitting from vegetated foreshores. Benefitting is defined by a required crest height reduction due to wave-vegetation interaction of at least 50 cm. For accuracy reasons, the required crest height reduction is set to zero for locations with a required crest height reduction of less than 25 cm and a significant wave height reduction of less than 10 cm and therefore excluded in the calculation of the mean required crest height reduction.

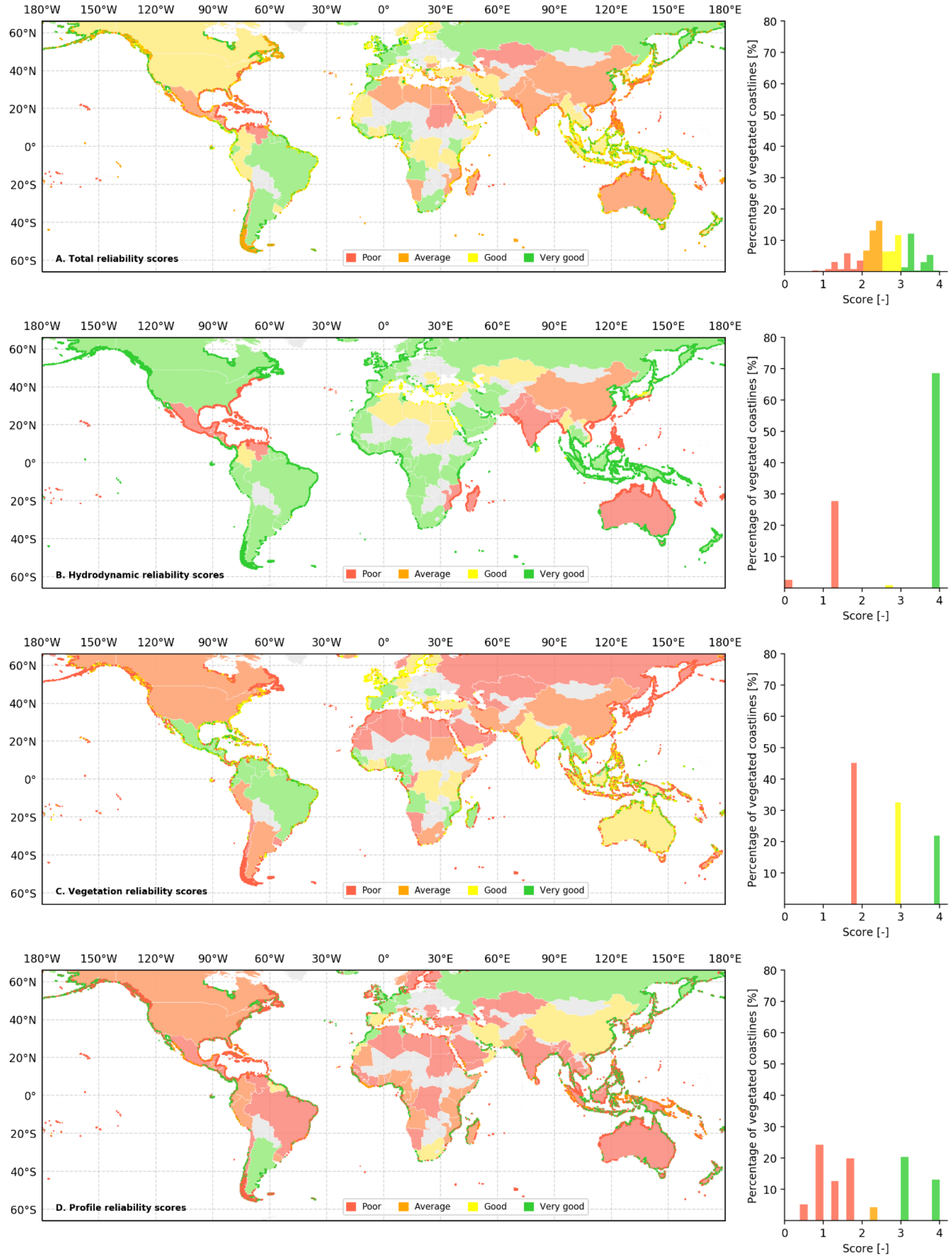

Supplementary Fig. 6 | Results reliability analysis. World maps showing geographically the results of **(A)** Total, **(B)** Hydrodynamic, **(C)** Vegetation and **(D)** Profile reliability scores. The indicators correspond to the following scores, poor (red) 0.0-2.0, average (orange) 2.0-2.5, good (yellow) 2.5-3.0, very good (green) 3.0 and higher. The histograms show the distribution of the reliability scores as percentage of the vegetated coastlines. Maps are created with Python 3.8.10 (https://python.org) using Cartopy (v0.18.0. Met Office UK. https://pypi.python.org/pypi/Cartopy/0.18.0), GeoPandas v0.8.1 (https://geopandas.org), Matplotlib v3.3.4 and GADM v2 (https://gadm.org) administrative boundaries.

Panel 1 – Performance at Atchafalaya Delta, LA, USA (m, LMSL)

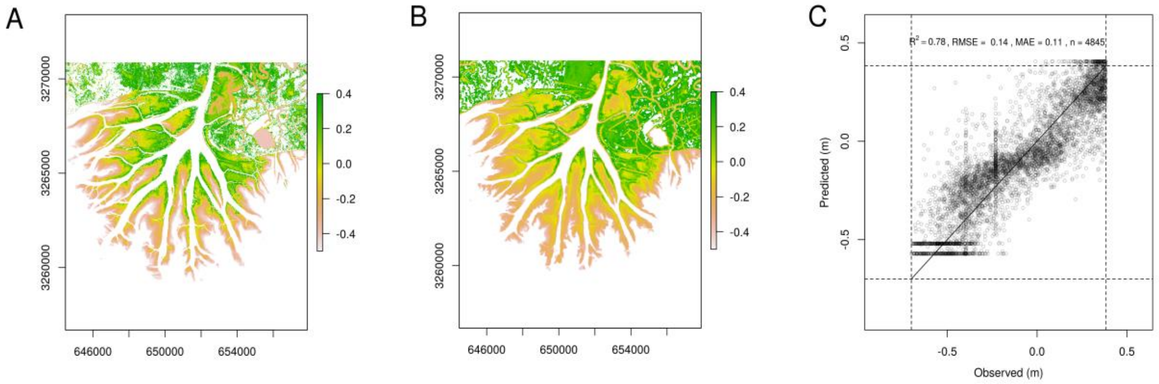

Panel 2 – Performance at Wachapreague, VA, USA (m, LMSL)

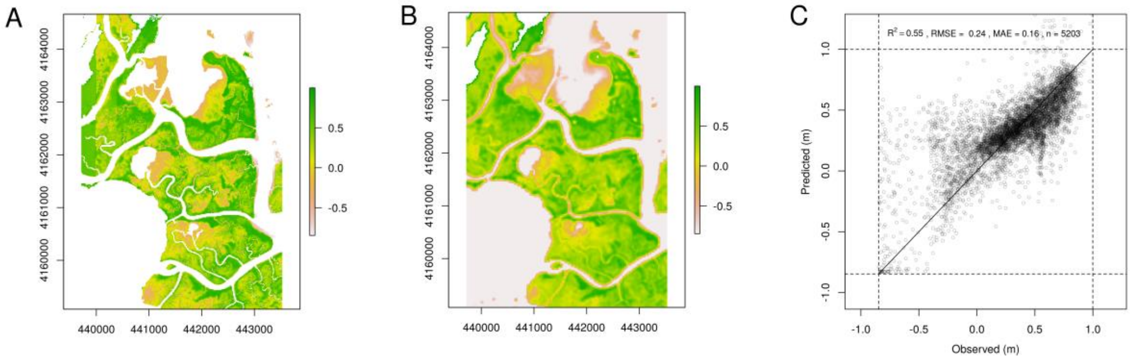

Panel 3 - Performance at FAST study sites (m, MSL)

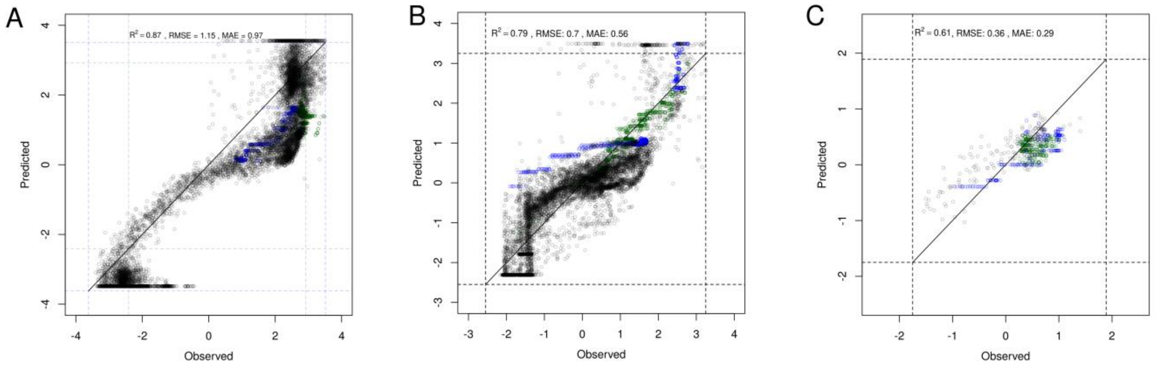

Supplementary Fig. 7 | Validation examples of the FAST intertidal elevation product In **Panel 1** the evaluation of the FAST intertidal map at Atchafalaya Delta, LA, USA. A) Observed elevation derived from the USGS Coastal National Elevation Database (CoNED) Project - Topobathymetric Digital Elevation Model (TBDEM, https://lta.cr.usgs.gov/coned_tbdem), B) predicted elevation, C) scatterplot of observed and predicted elevation.

In **Panel 2** evaluation at sites in Wachapreague, VA, USA A) Observed elevation derived from the USGS Coastal National Elevation Database (CoNED) Project - Topobathymetric Digital Elevation Model (TBDEM, https://lta.cr.usgs.gov/coned_tbdem), B) predicted elevation, C) scatterplot of observed and predicted elevation.

In **Panel 3** scatterplots showing observed versus predicted inter-tidal elevation (m, MSL) at the FAST case study sites in Europe. A) UK_1 and UK_2 on the east coast of the UK, B) NL_1 and NL_2 in the Westerschelde, SW Netherlands, C) ES_1 and ES_2 in Cádiz Bay, SW Spain. Green and blue points represent dGPS measurements (vertical accuracy of ± 0.02 m) from case study sites 1 and 2, respectively. Grey points are data derived from high resolution DTMs for the study sites in the UK and NL generated by local authorities (environment.data.gov.uk (UK), ahn.nl (NL) and UAV flights commissioned by the University of Cadiz(ES), bilinear re-sampled to 20m pixels. Dashed lines represent LAT and HAT values for the region. Solid line is the 1:1 relationship. Statistical measures of 'goodness-of-fit' are also shown.

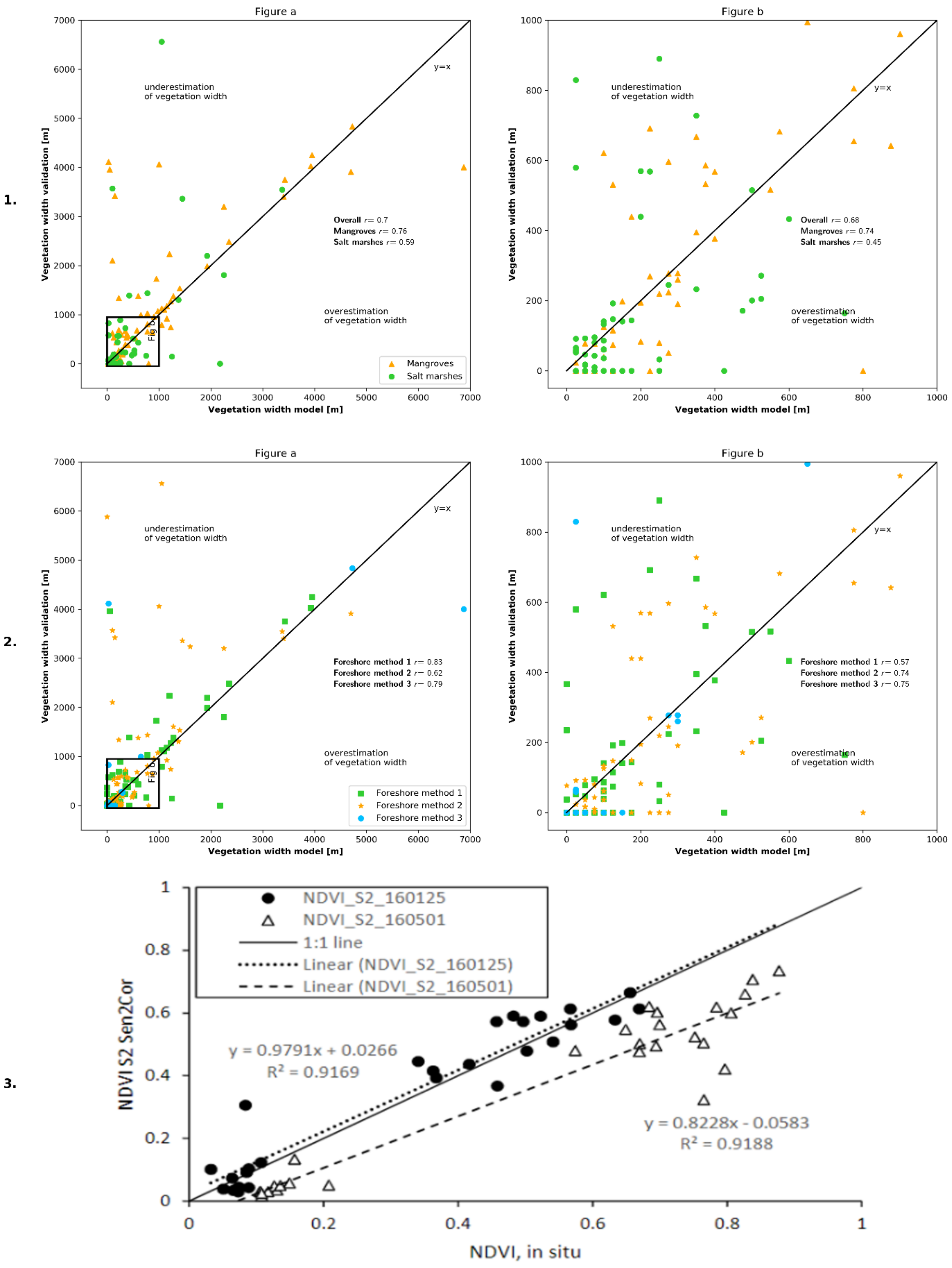

Supplementary Fig. 8 | Validation vegetation. Set of scatterplots with on the y-axis the vegetation width from the validation and on the x-axis the vegetation widths derived by the algorithm. The left plots cover a coastal vegetation belt width between 0 - 7000 metre and the right plots an extent between 0 -1000 metre. Vegetation width validation results divided based **(1)** on vegetation type, mangroves or salt marshes, **(2)** on foreshore derivation method, **(3)** NDVI validation of the FAST vegetation map based on field measurements at Zuidgors, The Netherlands taken in two different seasons (months January and May).

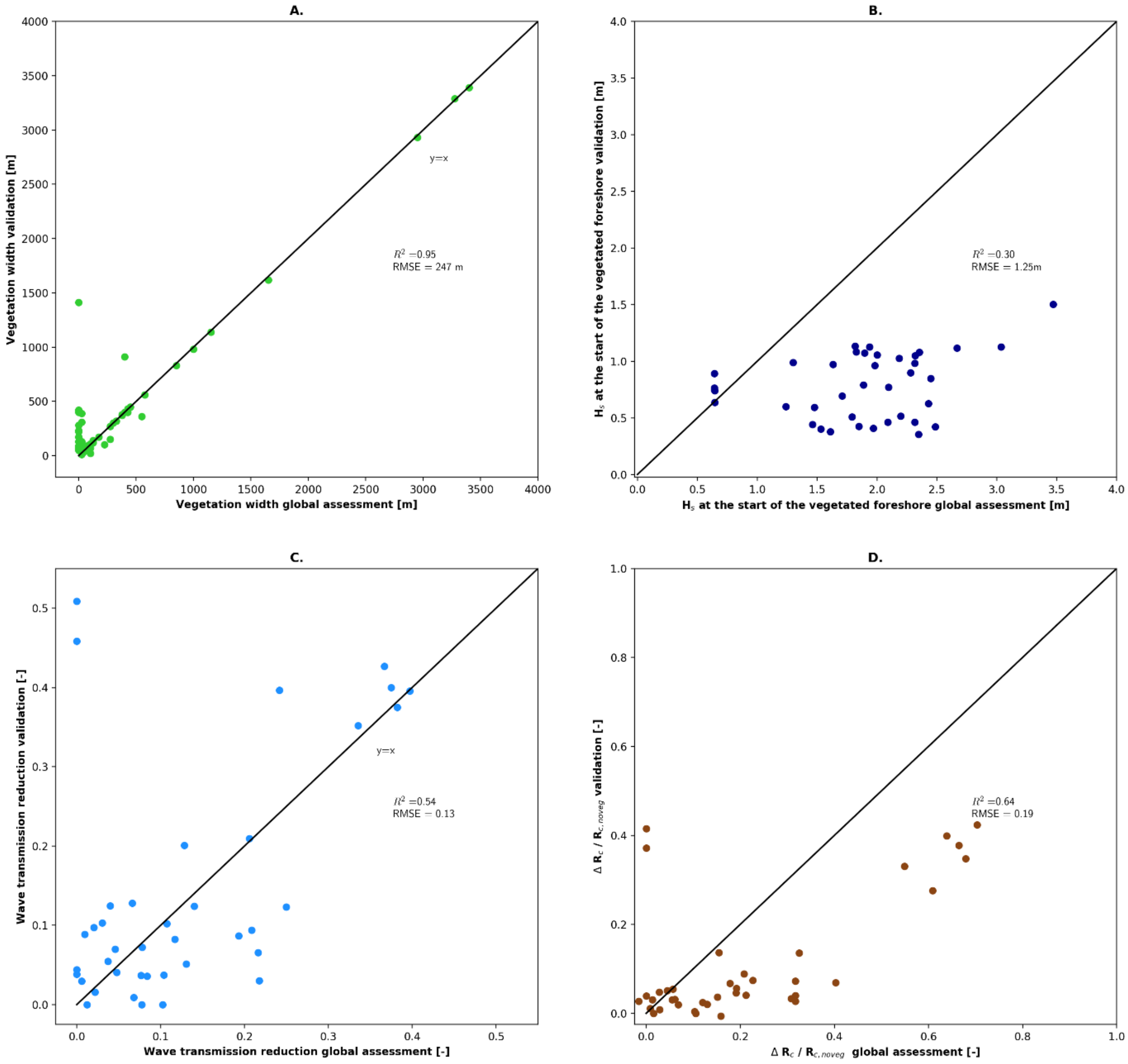

Supplementary Fig. 9 | Reduced wave transmission case study results for the Western Scheldt, The Netherlands. Figure showing the performance of the model used in the global assessment in comparison to a local analysis, using high-quality local data and numerical SWAN calculations to simulate offshore to nearshore wave propagation and XBeach calculations to simulate foreshore wave propagation. The vegetation width **(A)** is estimated accurately, R^2 = 0.95, during the global assessment, although smaller vegetation bands (<500m) are not always recognized. The case study area has a complex geometry and especially the most landward part of the estuary is sheltered from incoming waves. Using the depth limited approach for these locations induces a overestimation of the significant wave height at the start of the vegetated foreshore **(B)**. Despite this overestimation, the algorithm is able to approximate the wave transmission reduction **(C)** and the levee crest height reduction relative to the required crest height without vegetation presence **(D)** with reasonable accuracy.

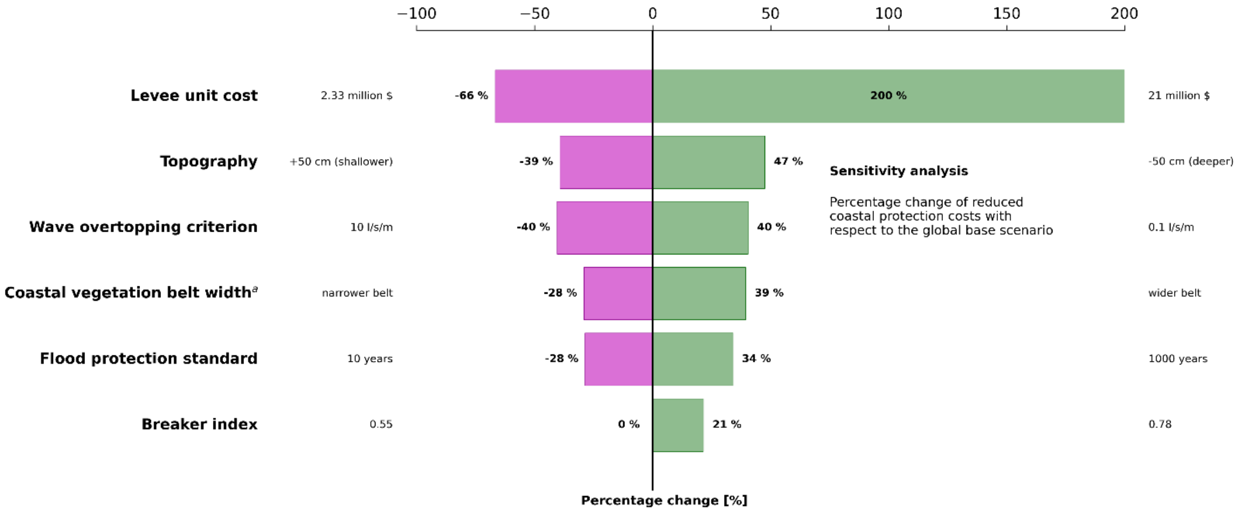

Supplementary Fig. 10 | Results sensitivity analysis. Bar plot indicating percentage change of reduced coastal protection costs with respect to the global base scenario for changes in levee unit cost, topography, wave overtopping criterion, coastal vegetation belt width, flood protection standard and wave breaker index.
